# Supplementary material for: Mechanisms governing the pioneering and redistribution capabilities of the non-classical pioneer PU.1
Source: Nat Commun. 2020 Jan 21;11:402. doi: 10.1038/s41467-019-13960-2 (PMC6972792; doi:10.1038/s41467-019-13960-2)
Supplement: Supplementary file 7 — Source data [file 41467_2019_13960_MOESM7_ESM.zip › Source_Data/FigureS2/FigureS2B_MotifScanOutput/KmeansCluster13/homerResults/motif20.similar.html]

motif20

## Information for motif20

A
T
G
C
A
G
T
C
T
C
G
A
C
T
A
G
A
G
T
C
A
G
T
C
C
G
T
A
C
G
T
A
A
G
C
T
A
T
G
C
T
C
G
A
  
Reverse Opposite:  

A
G
C
T
A
T
C
G
T
C
G
A
A
C
G
T
A
C
G
T
A
C
T
G
A
C
T
G
G
A
T
C
A
G
C
T
T
C
A
G
T
A
C
G
  

|  |  |
| --- | --- |
| p-value: | 1e-25 |
| log p-value: | -5.809e+01 |
| Information Content per bp: | 1.692 |
| Number of Target Sequences with motif | 115.0 |
| Percentage of Target Sequences with motif | 6.80% |
| Number of Background Sequences with motif | 936.5 |
| Percentage of Background Sequences with motif | 2.08% |
| Average Position of motif in Targets | 99.6 +/- 58.3bp |
| Average Position of motif in Background | 91.9 +/- 63.4bp |
| Strand Bias (log2 ratio + to - strand density) | 0.2 |
| Multiplicity (# of sites on avg that occur together) | 1.18 |
| Motif File: | file (matrix) reverse opposite |

### Similar de novo motifs found

|  |  |  |  |  |  |  |  |
| --- | --- | --- | --- | --- | --- | --- | --- |
| Rank | Match Score | Redundant Motif | P-value | log P-value | % of Targets | % of Background | Motif file |
| 1 | 0.905 | A T C G A G T C A C T G C T G A A C G T C G A T C T A G C T A G G T A C | 1e-23 | -53.341800 | 10.28% | 4.33% | motif file (matrix) |
| 2 | 0.882 | A G C T A T C G C G T A C G A T G C A T C T A G C T A G A G T C C A G T A T G C | 1e-13 | -31.054463 | 4.26% | 1.47% | motif file (matrix) |
